# Evaluation of Discoloration Removal by Polishing Resin Composites Submitted to Staining in Different Drink Solutions

**DOI:** 10.1155/2015/853975

**Published:** 2015-08-20

**Authors:** Denis Roberto Falcão Spina, João Ricardo Almeida Grossi, Rafael Schlögel Cunali, Flares Baratto Filho, Leonardo Fernandes da Cunha, Carla Castiglia Gonzaga, Gisele Maria Correr

**Affiliations:** Graduate Program in Dentistry, Positivo University, Rua Prof. Pedro Viriato Parigot de Souza, 5300 Campo Comprido, 81280-330 Curitiba, PR, Brazil

## Abstract

The aim of this study was to evaluate the discoloration effects of water, cola-based soft drink, coffee, and wine on resin composites used in restorative dentistry and the possibility of removing the stain with chair side manual polishing. The A2 shade of three materials was tested. Disc specimens were prepared. A spectrophotometer was used to measure the baseline CIE-Lab color parameters of each material (*n*=10) 24 hours after sample preparation. Samples were then immersed in a cola-based soft drink, coffee, or wine for 1 hour every day, for 30 days. For the remaining hours, the specimens were stored in distilled water. In the control group, the specimens were immersed in water for the whole period. The color differences (Δ*E*) were calculated after 7 and 30 days of storage, and after polishing with coarse Sof-Lex discs, and analyzed by two-way ANOVA with repeated measures and Tukey's HSD test (α=0.05). Luna presented higher Δ*E* values (3.41)^*a*^ followed by Durafill (2.82)^*b*^ and Herculite (2.24)^*c*^. For the drink solutions, Δ*E* values were higher for wine (4.40)^*a*^ followed by coffee (2.59)^*b*^ and for cola-based soft drink (2.23)^*c*^ and water (2.13)^*c*^ which were statistically similar. For time, Δ*E* values were higher for 30 days (3.97)^*a*^ and then for 7 days (2.48)^*b*^ and after polishing (2.04)^*c*^. The results indicate that color stability is material dependent. The types of drinks that patients consume also influence the color stability of restorative materials.

## 1. Introduction

The color stability of composite materials used in restorative dentistry is crucial, both after the material is completely cured and throughout the restoration's lifetime. Previous studies have described color changes occurring in direct dental composites [1, 2]; however, discoloration of resin-based materials may be caused by different factors [3].

Direct composite resins are composed of a polymer matrix and fillers and can be classified into nanocomposite, nanohybrid, microhybrid, and microfilled materials [4]. Chemical discoloration of these materials has been attributed to a change or oxidation in the amine accelerator, oxidation in the structure of the polymer matrix, and oxidation of the unreacted pendant methacrylate groups. Color stability can also depend on the interface of the matrix and the different fillers used in these materials [5, 6]. Thus, the color stability of direct composites can be affected by their chemical components.

Additionally, extrinsic factors can cause discoloration, for example, staining by absorption of colorants as a result of contamination from exogenous sources. The degree of discoloration from exogenous sources varies according to the eating and drinking habits of the patient. The effects of different drink solutions (e.g., coffee, wine, and cola-based soft drink) on the color stability of different restorative materials over time have been evaluated previously [1, 3].

Discoloration can be considered one of the main reasons for the replacement of esthetic restorations made with resin composite [7]. In general, the superficial layer of the restorations is the most affected by discoloration, since this is the one in contact with food and drink colorants, being more prone to extrinsic pigmentation. In some clinical situations, if the restoration is still in function but the material has been nonseverely stained, an attempt to avoid the premature replacement of a composite restoration in anterior teeth can be carried out. Depending on the depth of the stain, chair side repolish of the restoration could be a viable option to remove the superficial layer, attempting to recover its initial color or at least make it unnoticeable for the human eye [8–10].

The aim of this study was to evaluate the influence of different drink solutions on the color stability of different resin composites and the possibility of removing the stain with chair side manual repolishing. The following hypotheses were evaluated: (i) the type of resin composite evaluated may affect color stability; (ii) the type of drink solution might have an influence on color stability; and (iii) chair side repolishing would be effective in removing the stain.

## 2. Material and Methods

Disc specimens of three different methacrylate-based materials (Herculite Classic [Kerr Corporation, Orange, CA, USA], Durafill VS [Heraeus Kulzer GmbH & Co., Wehrheim, Germany], and Luna [SDI Ltd., Bayswater, Victoria, Australia]) (Table 1) were constructed with the aid of a polytetrafluorethylene matrix. All specimens were prepared under conditions of controlled humidity (55 ± 5%) and temperature (23 ± 1°C). Ten disk specimens, 8 mm in diameter and 1 mm in thickness, were prepared for each material.

The matrix was placed onto a microscope lamina of 1 mm in thickness, followed by the insertion of the resin composite. Next, a polyester strip was placed onto the orifice of the matrix and another microscope lamina of the same thickness was positioned on top of it. The resin composite was then light-cured for 40 seconds with a halogen curing unit (Optilux Demetron 400; Kerr Corporation) at a irradiance of 700 mW/cm^2^.

Subsequently, the color of the resin was evaluated using the CIE-Lab system with the aid of a spectrophotometer (VITA Easyshade 3D Master; Vita Zahnfabrik, Bad Säckingen, Germany), in order to define a baseline color measurement. The specimens were then placed in water, cola-based soft drink, coffee, or wine for 1 hour every day, for 30 days. For the remaining hours, the specimens were stored in distilled water. Their color parameters were then evaluated after 7 and 30 days. After 30 days, the specimens were polished with coarse Sof-Lex disc and again submitted to color evaluation. In the control group, the specimens were immersed in water for the whole period.

The CIE *L* ^*∗*^ parameter corresponds to the degree of lightness and darkness, whereas *a* ^*∗*^ and *b* ^*∗*^ coordinates correspond to red or green (+*a* ^*∗*^ = red, −*a* ^*∗*^ = green) and yellow or blue (+*b* ^*∗*^ = yellow, −*b* ^*∗*^ = blue), respectively. These coordinates were used to calculate the color difference (Δ*E*) between the baseline color measurement (24 hours after photo-curing) and color measurements after storage for 7 and 30 days and after polishing. Before each color measurement, the immersed specimens were dried with absorbent paper. The Δ*E* for each experimental time was calculated using the following:

(1) $$\begin{array}{c} \Delta E={\left[\;{\left(\;\Delta L^{*}\;\right)}^{2}+{\left(\;\Delta a^{*}\;\right)}^{2}+{\left(\;\Delta b^{*}\;\right)}^{2}\;\right]}^{1/2}. \end{array}$$

In which Δ*L* ^*∗*^, Δ*a* ^*∗*^, and Δ*b* ^*∗*^ represent the differences between the readings of the color parameters obtained from the specimens under the different conditions evaluated. The results of Δ*E* were analyzed by two-way ANOVA with repeated measures and Tukey's HSD test (*α* = 0.05).

## 3. Results

The mean and standard deviation values of Δ*E* are presented in Table 2. Statistical analysis indicated significant differences for all the main factors, double and triple interactions (*p* = 0.000).

Considering the individual factors, Luna presented higher Δ*E* values (3.41)^*a*^, followed by Durafill VS (2.82)^*b*^ and Herculite (2.24)^*c*^. For the drink solutions, Δ*E* values were higher for wine (4.40)^*a*^, followed by coffee (2.59)^*b*^, and for cola-based soft drink (2.23)^*c*^ and water (2.13)^*c*^, which were statistically similar. For time, Δ*E* values were higher for 30 days (3.97)^*a*^ and then for 7 days (2.48)^*b*^ and after polishing (2.04)^*c*^.

## 4. Discussion

The three hypotheses evaluated in the present study were accepted. There were significant differences between the resin composites evaluated, and significant effects of the type of drink solution and time on color stability were detected. Also, chair side polishing significantly reduced the color difference, when compared to the values obtained after staining for 30 days in the different drink solutions.

Discoloration can be evaluated visually or by instrumental techniques. Shade selection with visual guides can be considered a subjective method, while shade obtained with spectrophotometers can be thought of as an objective method. In the current study, the system used for measuring chromaticity was chosen to record color differences because it is more precise than visual techniques and is capable of detecting color differences below the threshold of visual perception [11, 12].

Storage in water is used frequently as a model of in vitro aging. It is well known that water absorbed by the polymer matrix causes filler matrix debonding and hydrolytic degradation of the filler [13–16]; however, it can also change the refractive index of the material [17]. Therefore, an increase in Δ*E* values might be affected by water absorption occurring in the resin composites used. In the present study, the Δ*E* values showed a tendency to increase as immersion period increased, suggesting that the color of resin composites would tend to darken over long-term clinical use [17]. Cola-based soft drinks, coffee, and wine are commonly consumed drinks and other investigators have demonstrated discoloration of composite materials upon exposure to these solutions [2, 18–20]. In the present study, there was no difference in discoloration between exposure to water and to cola-based soft drink. This is in accordance with other studies, because cola drinks do not appear to be strongly implicated in color change of composites, despite the presence of phosphoric acid [9, 21]. It is likely that the lower pH of the drinks tested affected the composite resin surface, increasing pigment absorption. However, the alcohol in wine increased this color degradation even further.

In the current study, color variation was considered imperceptible when <1, clinically acceptable when ≤3.3, and unacceptable when higher than 3.3. After aging, all specimens showed changes in the *L* ^*∗*^, *a* ^*∗*^, and *b* ^*∗*^ coordinates, but all of the color differences were deemed acceptable. The mean color difference (Δ*E*) was the highest in Luna resin composite (Δ*E* = 3.41), the lowest in Herculite resin composite (Δ*E* = 2.24), and intermediate in Durafill VS resin composite (Δ*E* = 2.82). This suggests that more color degradation occurred in the nanohybrid material than in the microfilled and microhybrid materials.

Degradation of residual amines and oxidation of residual unreacted carbon-carbon double bonds culminate in the formation of yellowing compounds in all resin-based materials [22, 23]. Durafill VS and Luna are microfilled and nanohybrid resin composites and have a small filler size. This might be expected to contribute to decreased staining and enhanced esthetic appearance [24]. However, composites with a smaller filler size do not necessarily show low levels of discoloration, as demonstrated in the present study. Staining of composite resins is dependent on monomer structure, as well as surface irregularities. Hence, the results of the present study are in agreement with previous findings.

As mentioned before, stained resin composite restorations are a common finding in the dental practice. In this way, the present study attempted to determine the superficial staining in three resin composites because it was expected that staining would occur in the most external layer of the material [8, 25]. Therefore, polishing the resin composite after the staining procedures would possibly remove discoloration. The results indicated that polishing of the specimens' surface stained by the drink solutions was able to significantly reduce the Δ*E* values for the three tested composite resins, especially when stored in wine and coffee. In the groups immersed in water and cola-based soft drink, Δ*E* values were already lower than 2 in all times. Also, it is important to note that after polishing, Δ*E* values were considered clinically acceptable, being lower than 2.36 in all groups, except for Durafill VS (storage in wine), where Δ*E* was 4.09. Regarding storage in wine, which was considered the most staining of the drink solutions used in the present study, Luna showed a greater decrease in the Δ*E* values after polishing and a reduction of approximately 80% of discoloration, followed by Herculite with a reduction of 55% and Durafill VS with a 40% reduction in discoloration. Similar percentages of reduction in discoloration after polishing were also reported in the literature [9]. It is important to note that this difference between stained specimens' and polished specimens' color showed a good quality of polishing procedures. Also, the nature of the stain was an important factor influencing the level of the reduction in discoloration [10].

Many materials with different compositions are available on the market. More studies should be conducted in order to evaluate the color changes of resin composites in different situations. This would aid in understanding and improve esthetic results in the long term [26].

## 5. Conclusions

In the present study, all the materials tested showed visible color changes after immersion in drink solutions. The wine solution used caused the most staining. Luna resin composite showed a significantly higher color change as compared to the other resins investigated. Chair side polishing of the specimens' surface stained by the drink solutions was able to significantly reduce the Δ*E* values for the tested materials, especially after being stored in wine and coffee.

## Figures and Tables

**Table 1:** Name, material type, and manufacturer of the resin composites tested.

Product/manufacturer	Material type	Composition
Herculite Classic (Kerr Corporation, Orange, CA, USA)	Microhybrid composite	BIS-GMA, TEGDMA, camphorquinone, amine, iron oxide pigments, aluminum borosilicate glass, colloidal silica.
Durafill VS (Heraeus Kulzer GmbH & Co., Wehrheim, Germany)	Microfilled composite	BisGMA/TEGDMA, UDMA, silicon dioxide, splinter polymer (66% by volume)
Luna (SDI, Bayswater, Victoria, Australia)	Nanohybrid composite	Multifunctional methacrylate ester and inorganic filler (61% by volume)

**Table 2:** Mean ± standard deviation values of Δ*E* for all conditions tested. Values followed by the same letters are statistically similar (*p* > 0.05).

Material	Solution	Time	Δ*E*
Herculite Classic	Water	7 days	1.37 ± 0.36^ab^
		30 days	1.83 ± 0.35^abc^
		After polishing	1.59 ± 0.39^ab^
	Cola	7 days	1.61 ± 0.32^ab^
		30 days	1.97 ± 0.73^abc^
		After polishing	1.73 ± 0.51^abc^
	Coffee	7 days	1.33 ± 0.22^ab^
		30 days	2.68 ± 0.71^cdefg^
		After polishing	2.09 ± 0.48^abcde^
	Wine	7 days	3.11 ± 0.72^defgh^
		30 days	5.30 ± 1.36^k^
		After polishing	2.36 ± 0.53^bcdef^

Durafill VS	Water	7 days	1.18 ± 0.79^a^
		30 days	2.36 ± 0.95^bcdef^
		After polishing	2.11 ± 0.39^abcde^
	Cola	7 days	1.68 ± 0.56^abc^
		30 days	2.27 ± 0.23^bcdef^
		After polishing	1.85 ± 0.36^abc^
	Coffee	7 days	1.83 ± 0.44^abc^
		30 days	3.81 ± 0.50^hij^
		After polishing	2.27 ± 0.72^bcdef^
	Wine	7 days	4.28 ± 0.51^ijk^
		30 days	6.78 ± 1.03^l^
		After polishing	4.09 ± 0.82^hij^

Luna	Water	7 days	3.06 ± 0.57^defgh^
		30 days	3.59 ± 0.35^ghi^
		After polishing	2.12 ± 0.57^abcdef^
	Cola	7 days	3.16 ± 0.47^fgh^
		30 days	4.38 ± 0.37^ijk^
		After polishing	1.47 ± 0.41^ab^
	Coffee	7 days	3.04 ± 0.56^defgh^
		30 days	4.85 ± 0.51^jk^
		After polishing	1.45 ± 0.40^ab^
	Wine	7 days	4.26 ± 0.62^ij^
		30 days	8.07 ± 0.53^m^
		After polishing	1.55 ± 0.67^ab^
